# The energy blockers bromopyruvate and lonidamine lead GL15 glioblastoma cells to death by different p53-dependent routes

**DOI:** 10.1038/srep14343

**Published:** 2015-09-21

**Authors:** Magdalena Davidescu, Lara Macchioni, Gaetano Scaramozzino, Maria Cristina Marchetti, Graziella Migliorati, Rita Vitale, Angela Corcelli, Rita Roberti, Emilia Castigli, Lanfranco Corazzi

**Affiliations:** 1 Department of Experimental Medicine, University of Perugia, Perugia, Italy; 2 Department of Chemistry, Biology and Biotechnology, University of Perugia, Perugia, Italy; 3 Department of Medicine, University of Perugia, Perugia, Italy; 4 IMM-CNR, Institute for Microelectronics and Microsystems, National Research Council, Lecce, Italy; 5 Department of Basic Medical Sciences, Neuroscience and Sensory Organs, University of Bari “A. Moro”, Bari, Italy

## Abstract

The energy metabolism of tumor cells relies on aerobic glycolysis rather than mitochondrial oxidation. This difference between normal and cancer cells provides a biochemical basis for new therapeutic strategies aimed to block the energy power plants of cells. The effects produced by the energy blockers bromopyruvate (3BP) and lonidamine (LND) and the underlying biochemical mechanisms were investigated in GL15 glioblastoma cells. 3BP exerts early effects compared to LND, even though both drugs lead cells to death but by different routes. A dramatic decrease of ATP levels occurred after 1 hour treatment with 3BP, followed by cytochrome c and hexokinase II degradation, and by the decrease of both LC3I/LC3II ratio and p62, markers of an autophagic flux. In addition, Akt(Ser^473^) and p53(Ser^15^/Ser^315^) dephosphorylation occurred. In LND treatment, sustained ATP cellular levels were maintained up to 40 hours. The autophagic response of cells was overcome by apoptosis that was preceded by phosphatidylinositol disappearance and pAkt decrease. This last event favored p53 translocation to mitochondria triggering a p53-dependent apoptotic route, as observed at 48 and 72 hours. Adversely, in 3BP treatment, phospho-p53 dephosphorylation targeted p53 to MDM2-dependent proteolysis, thus channeling cells to irreversible autophagy.

Glioblastoma tumors arising from glial cells are the most common and aggressive primary brain tumors. Human glioblastomas display high apoptosis resistance mediated by multiple deregulated signaling pathways^1^,^2^. Recent literature points to the highly glycolytic character of glioblastoma cells as a key mediator in their apoptosis resistance^3^,^4^. This line of thought derives from the original observation of Otto Warburg nearly 90 years ago and is confirmed by considerable information acquired in the last decades about the molecular mechanisms that are involved. The increased dependence on glycolysis for ATP generation of tumor versus normal cells has provided new therapeutic approaches that selectively attack tumor cells. The high glucose consumption is supported by type II hexokinase (HK-II) overexpression; its binding to mitochondria through interaction with a voltage-dependent anion channel (VDAC1) hinders cytochrome c (cyt c) and apoptosis inducing factor (AIF) release in the cytosol resulting in cell death suppression^5^. This finding stimulated approaches aimed to break HK-II/VDAC1 association.

The potent HK-II inhibitor bromopyruvate (3BP) is an energy blocker and a promising agent for cancer therapy^6^. 3BP is an alkylating agent that reacts with thiol and hydroxyl groups of several enzymes and is able to induce cell death in tumor cell lines through a variety of biochemical mechanisms^7^,^8^,^9^,^10^,^11^. In addition, 3BP causes regression of solid tumors by ATP depletion^12^. The therapeutic agent lonidamine (LND), first synthesized and designed as an antispermatogenic drug^13^, is an antiglycolytic that acts by inhibiting the mitochondria bound HK-II. Contrarily to 3BP, LND contains low structural reactivity since it is not reactive towards -SH groups. Though the molecular mode of action is elusive, LND targets mitochondria and induces apoptosis via a direct effect on the PT pore^14^. In a model of brain tumor, the mechanism of action of LND involves inhibition of lactate efflux and intracellular acidification^15^. When tested on temozolomide-resistant glioma cells, LND, used as a cytotoxic drug in mitochondria-directed chemotherapy, triggers apoptosis as principal death modality^16^.

GL15 glioblastoma cells are tumorigenic and highly invasive in *in vivo* experimental models^17^ and exhibit deregulated signaling pathways typical of human glioblastomas^18^,^19^,^20^,^21^. GL15 cells can be driven to metabolic decline by different strategies. An apoptotic pathway was induced following an imbalanced control of cell cycle progression or the alteration of cardiolipin (CL) synthesis in palmitate-treated cells^19^. An autophagic process, severe ATP reduction, and viability loss were triggered by 3BP^22^.

Considering the emerging relevance of 3BP as an antitumor drug, we thought it would be of interest to unravel the biochemical mechanisms underlying 3BP effects in GL15 glioblastoma cells compared to antiglycolytic LND. We found that 3BP exerts very early effects, compared to LND, both drugs leading cells to death, although by different routes. In GL15 cells, 3BP targets p53 to dephosphorylation and degradation, thus favoring an irreversible autophagic process. Adversely, LND orients GL15 cells towards autophagy that is eventually overcome by apoptosis, favored by p53 translocation to mitochondria.

## Results

### Contribution of glycolysis and OXPHOS to ATP levels in GL15 cells

**Figure 1:**
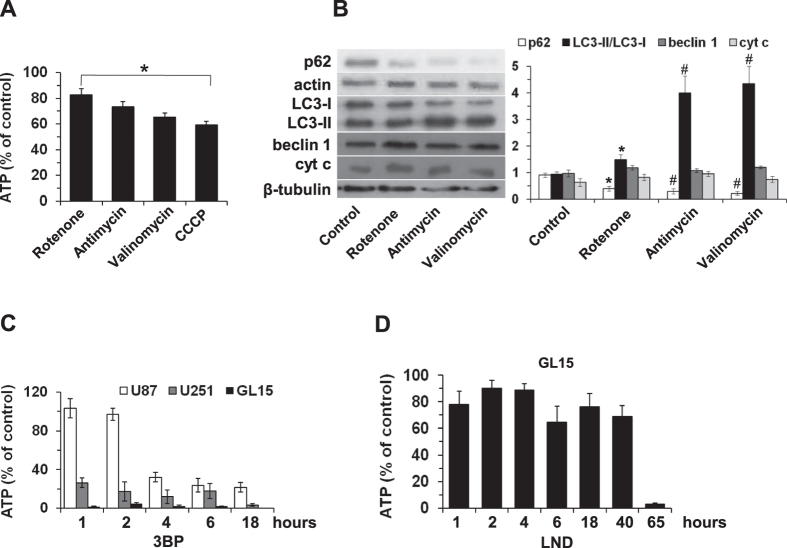
Effect of respiratory chain inhibitors, and 3BP and LND energy blockers on ATP levels in glioblastoma cells. (**A**,**B**) GL15 cells were incubated for 6 hours with the mitochondrial inhibitors rotenone (10 μM), antimycin A (25 μM), valinomycin (2 μM), or CCCP (10 μM). (**A**) ATP levels as the contribution of glycolysis and OXPHOS. (**B**) Expression of cyt c and the autophagic proteins p62, LC3, and beclin 1. Expression levels were analyzed by densitometry. p62 is referred to actin. LC3 is shown as LC3-II/LC3-I ratio. Beclin 1 and cyt c are referred to β-tubulin. Data are the mean ± SD of three independent experiments (^#^p < 0.01, and *p < 0.05 vs control). In (**B**), a representative blot is shown. (**C**) U87, U251, and GL15 glioblastoma cells were incubated with 80 μM 3BP. (**D**) GL15 cells were incubated with 400 μM LND for the indicated times. After the treatments, cells were recovered and ATP was determined. Data are the mean ± S.D. of four independent experiments. In control cells, ATP levels did not vary significantly along the incubation times (GL15, 3.73 ± 0.99 nmol/well; U87, 3.30 ± 0.62 nmol/well; U251, 3.78 ± 0.45 nmol/well).

Cells produce ATP through two mechanisms, glycolysis and mitochondrial oxidative phosphorylation (OXPHOS). Their contribution to ATP levels in GL15 cells was evaluated by treating cells with mitochondrial inhibitors (Fig. 1A). Rotenone produced about 17% decrease of ATP cellular levels, corresponding to the contribution of complex I-feeding respiratory substrates. In the presence of the downstream inhibitor antimycin, the ionophore valinomycin, and the uncoupler CCCP, ATP levels decreased by 27–41% of control, suggesting that this range represents the contribution of mitochondrial OXPHOS to ATP synthesis. The inhibition of mitochondrial ATP production was accompanied by an autophagic process, as evidenced by an increase of the truncated, phosphatidylethanolamine (PE)-conjugated LC3 protein (LC3-II). Indeed, the LC3-II/LC3-I ratio was about 1 in the control and increased with rotenone and even more so with valinomycin and antimycin A, due to a shift of LC3-I towards LC3-II. This process was paralleled by the decrease of p62 (Fig. 1B). It is worth noting that the mitochondrial inhibitors did not influence significantly beclin 1 and cyt c expression.

### Influence of 3BP and LND on ATP levels in glioblastoma cells

After 1 hour incubation of GL15 cells with 80 μM 3BP, ATP levels decreased dramatically (Fig. 1C), indicating the inhibition of ATP synthesis in both mitochondrial and cytosolic compartments. Similarly, 80 μM 3BP treatment decreased ATP levels in U251 glioblastoma cells, whereas the kinetics of ATP disappearance was much slower in U87 glioblastoma cells (Fig. 1C). Treatment of GL15 cells with 400 μM LND for up to 40 hours did not influence significantly ATP levels, which collapsed after only 65 hours (Fig. 1D).

### Effects of 3BP and LND on the expression of autophagic and mitochondrial proteins

**Figure 2:**
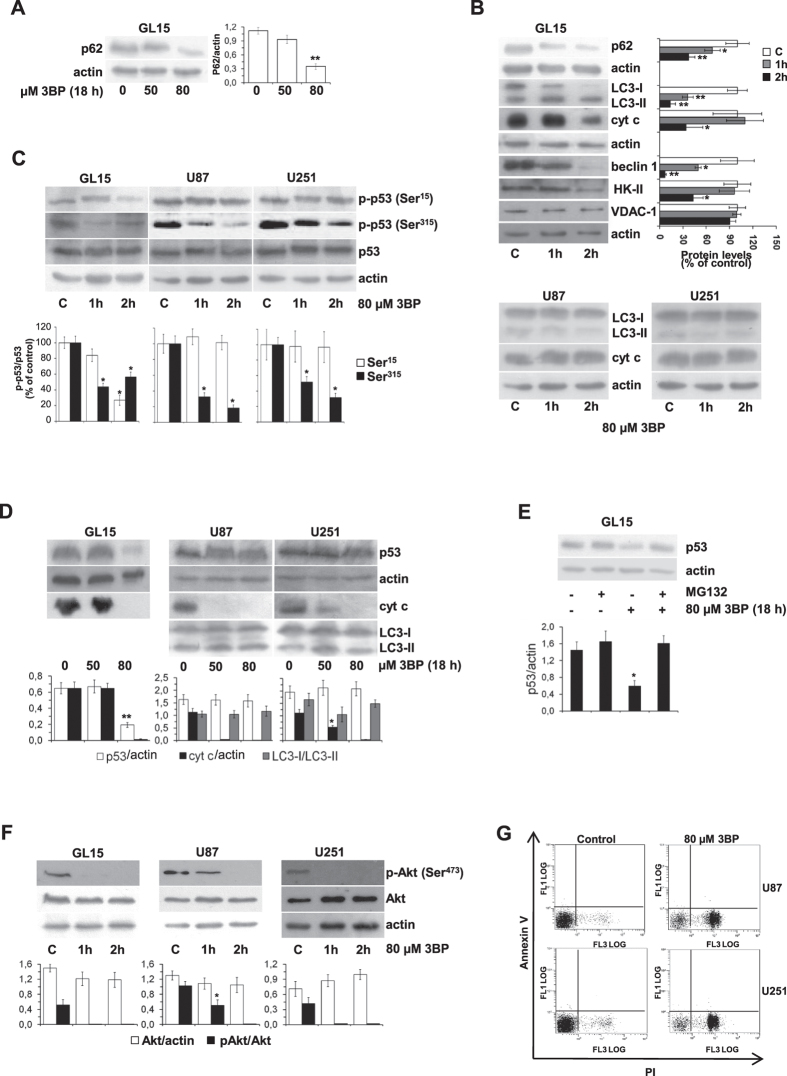
3BP exerts different effects in GL15 and U87/U251 glioblastoma cells. Cells were incubated with increasing 3BP concentrations for 18 h (**A**,**D**), or with 80 μM 3BP up to 2 hours (**B**,**C**,**F**), or with 80 μM 3BP for 18 hours (**E**). Protein expression was evaluated by Western blotting and analyzed by densitometry. (**B**) LC3-I/LC3-II ratio is expressed as percent of control; the other proteins are referred to the respective actin. (**C**) p53 protein expression and phosphorylation status at Ser-15 and Ser-315. The ratio p-p53/p53 is expressed as percent of control. (**D**) LC3I/LC3II ratio is reported, the other proteins are referred to actin. (**E**) Control and 3BP-treated GL15 cells were incubated in the absence or presence of the proteasome inhibitor MG132. (**F**) Akt phosphorylation status. In each of (**A–F**) panels, data are the mean ± SD of three independent experiments (**p < 0.01, and *p < 0.05 vs control). Representative blots are shown. (**G**) U87 and U251 cells were incubated with 80 μM 3BP for 18 hours, resuspended in isotonic buffer, stained with Annexin V-FITC and PI, and analyzed by flow cytometry (10,000 events). The dot plot distribution of particles reporting Annexin V-FITC fluorescence (FL1) versus PI fluorescence (FL3) shown is representative of three independent experiments.

We have shown previously that treatment of GL15 cells with 80 μM 3BP for 18 hours triggers an autophagic process, as demonstrated by the conversion of LC3 to the lipidated LC3-II form^22^. The autophagic flux is confirmed by a significant decrease of p62 (Fig. 2A). To investigate short-term effects of 3BP, GL15 cells were treated with 80 μM 3BP for 0–2 hours and the expression of p62, LC3, beclin 1, and mitochondrial proteins was evaluated by Western blotting. The conversion of LC3 protein to the lipidated LC3-II form was clear already after 1 hour incubation with 3BP, with the LC3-I to LC3-II ratio decreasing dramatically within 2 hours of incubation. A parallel decrease was observed for p62 and beclin 1 expression, whereas cyt c and HK-II decreased after 2 hours, and VDAC1, an integral protein of the inner mitochondrial membrane, did not change (Fig. 2B). qRT-PCR analysis of beclin 1, cyt c, and HK-II after 2 hour treatment with 3BP indicated that mRNA levels were not altered (not shown).

A relation between LC3-I to LC3-II conversion and p53 activity has been reported^23^. Therefore, we analyzed 3BP effects on the p53 status in GL15 cells and, for comparison, in U87 and U251 glioblastoma cell lines. No variations of p53 total protein were observed within 2 hour 3BP treatment in any of the tested cell lines (Fig. 2C). The p53 phosphorylation at Ser-15 decreased in GL15 cells after 2 hours, but remained unchanged in U87 and U251 cells. Adversely, phosphorylation at Ser-315 decreased in each of the cell lines (Fig. 2C). Dephosphorylation of p53(Ser-15) is predictive of degradation, via interaction with MDM2^24^. Indeed, a decrease of p53 was observed after 18 hour treatment with 80 μM 3BP in GL15 cells that was reversed by the proteasoma inhibitor MG132 (Fig. 2D,E). According to p-p53(Ser-15) status, no p53 decrease was found in U87 and U251 cells (Fig. 2D).

As previously observed in GL15 cells^22^ and confirmed in this study, 3BP produced Akt dephosphorylation also in U87 and U251 glioblastoma cell lines (Fig. 2F). Contrarily to GL15, where Akt inactivation could be predictive of the observed autophagic route, no autophagic response was observed in U87 and U251 cells (Fig. 2B,D). The different behavior of U87 and U251 versus GL15 cells was confirmed by FACS analysis that showed an increase of PI fluorescence, while Annexin V fluorescence was unchanged, indicating a necrotic process (Fig. 2G).

**Figure 3:**
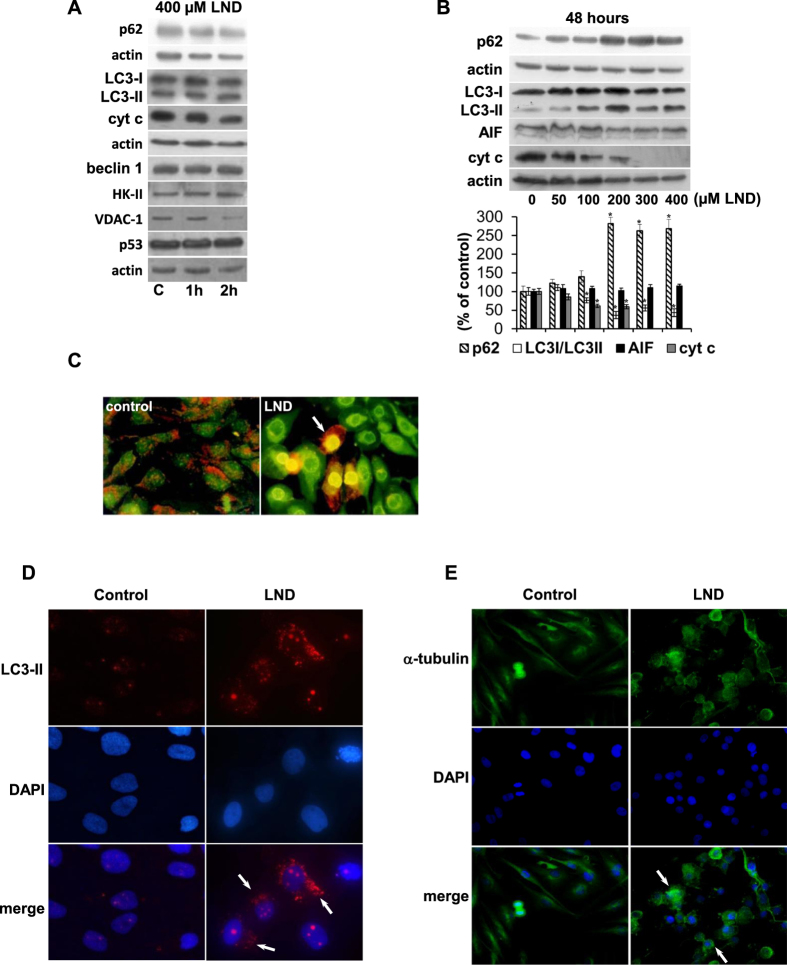
Autophagic response of GL15 cells to LND. Cells were incubated with 400 μM LND for 1–2 hours (**A**) or with 50–400 μM LND for 48 hours (**B**). Protein expression was evaluated by Western blotting and analyzed by densitometry. Data are the mean ± SD of three independent experiments (*p < 0.05 vs control). Representative blots are shown. (**C**) Cells were treated with 400 μM LND for 48 hours and stained with acridine orange for 15 min. The arrow indicates acidic vesicular organelles. Original magnification 400X. (**D**) Cells were treated with 400 μM LND for 48 hours and immunostained with a LC3 antibody. The arrows indicate bright puncta. Original magnification 600X. (**E**) Cells were treated with 400 μM LND for 48 hours and immunostained with an α-tubulin antibody. LND caused cytoskeleton disorganization. Arrows indicate α-tubulin condensed in the perinuclear zone. Original magnification 400X.

The treatment of GL15 cells with 400 μM LND for up to 2 hours did not affect p62, LC3, and beclin 1 autophagic proteins, as well as p53 and mitochondrial proteins (Fig. 3A). The long-term effects of LND were investigated by incubating GL15 cells with increasing LND concentrations. An increase in LC3-II protein with 100–400 μM LND was observed at 48 hours (Fig. 3B). At 300 and 400 μM LND, cyt c disappeared, whereas AIF did not change significantly in any conditions. It is worth noting that p62 expression increased at 200–400 μM LND (Fig. 3B). An autophagic scenario was evidenced by acridine orange staining that showed acidic vesicular organelles in LND-treated cells, whereas control cells had the classical punctuate orange staining of acidic compartments. Moreover, after LND treatment a diffuse acidification was evident in cells presenting autophagolysosomes (Fig. 3C). A further evidence that LND induces autophagy in GL15 cells was obtained by immunofluorescence analysis of LC3 that, when localized in autophagosome membranes, appears as bright puncta. The number and intensity of punctuate LC3 fluorescence increased after 48 hours of LND treatment (Fig. 3D). We found that LND induces a cellular phenotype in GL15 cells characterized by cytoplasm transparency, although with apparent integrity of the nuclear membrane (not shown). For this reason we evaluated the cytoskeleton integrity by immunofluorescence using an α-tubulin antibody. After LND treatment (400 μM, 48 hours) cytoskeleton was dramatically disorganized and abnormally condensed in the perinuclear zone (Fig. 3E).

**Figure 4:**
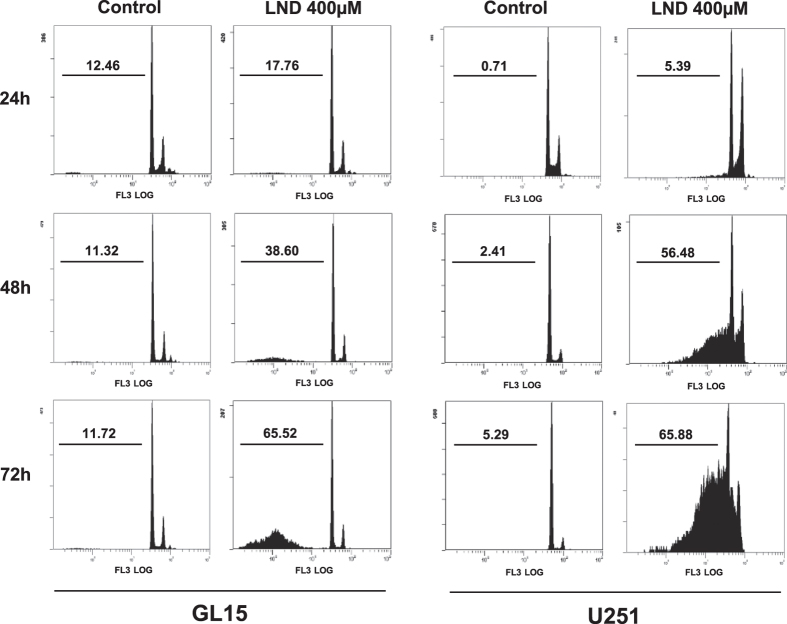
LND induces apoptotic cell death in glioblastoma cells. Cells were incubated with 400 μM LND for the indicated times and the percent of apoptotic cells (numbers in each plot) was evaluated by flow cytometry analysis of fragmented DNA after PI staining in hypotonic solution. The results shown are representative of three independent experiments.

**Figure 5:**
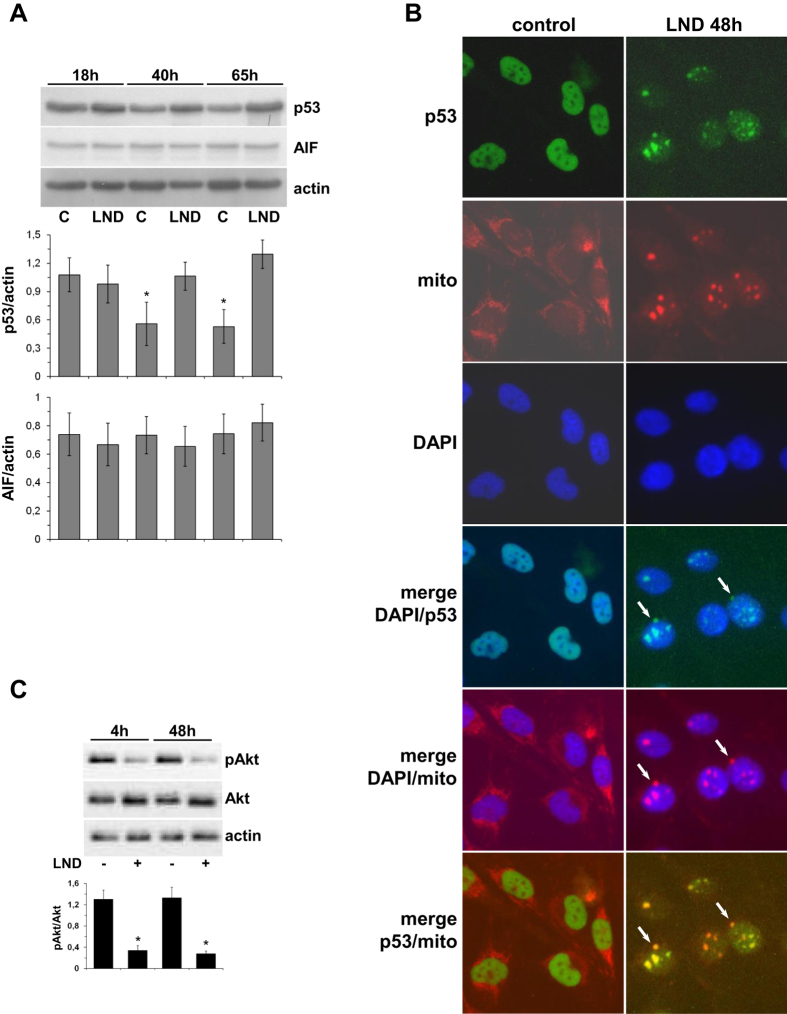
Effect of LND on p53, AIF, and Akt in GL15 cells. Cells were treated with 400 μM LND for the times indicated in each panel. (**A**) p53 and AIF proteins expression was evaluated by Western blot analysis of total cell lysates. For each protein, the ratio relative to actin was calculated by densitometric analysis. Data are the mean ± SD of three independent experiments (*p < 0.05 vs control). Representative blots are shown. (**B**) Immunofluorescence analysis shows that LND modifies the cellular localization of p53. The arrows indicate co-localization of p53 with mitochondria aggregated in the perinuclear zone in LND-treated cells. Images are representative of three experiments. (**C**) LND favors Akt dephosphorylation. Total Akt and pAkt expression was evaluated by Western blot analysis. pAkt/Akt was calculated by densitometric analysis. Data are the mean ± SD of three independent experiments (*p < 0.01). A representative blot is shown.

Since cytoskeleton alterations, as well as an increase of p62 expression in autophagic cells could predict the induction of an apoptotic route^25^,^26^ we investigated a possible evolution of GL15 cells towards apoptosis. FACS analysis indicated that no DNA fragmentation occurred in cells treated with 400 μM LND for 24 hours. However, when the treatment was prolonged for 48 and 72 hours about 39% and 65% apoptotic cells were found, respectively. This trend was confirmed in U251 cells (Fig. 4). No caspase-3 activity was detected in GL15 cells after 48 and 72 hours of LND treatment (not shown), thus excluding the involvement of the cyt c cascade in the onset of the apoptotic process. The expression of p53 and of the caspase-independent pro-apoptotic mitochondrial protein AIF were not affected by LND. However, for prolonged incubation times control cells showed a significant p53 decrease (Fig. 5A).

### LND modifies the cellular localization of p53 in GL15 cells

The binding of the tumor suppressor p53 to mitochondria promotes apoptosis^23^. To evaluate whether LND could exert a pro-apoptotic effect by favoring the interaction between p53 and mitochondria, we analyzed the cellular localization of p53 in GL15 cells after 48 hours with 400 μM LND (Fig. 5B). Diffuse nuclear localization of p53, with the exclusion of nucleoli, and uniform cytoplasmic distribution of mitochondria were observed in control cells. In LND-treated cells, p53 co-localized with mitochondria, which appeared aggregated in the perinuclear zone. Since p53 translocation to mitochondria is connected to PI3 Kinase/Akt signaling^27^, we analyzed Akt, which is constitutively phosphorylated in GL15 cells^20^. After treatment of cells with 400 μM LND, Akt activity was significantly inhibited as demonstrated by the decrease of pAkt at 4 and 48 hours (Fig. 5C).

### Analysis of GL15 cell lipids

**Figure 6:**
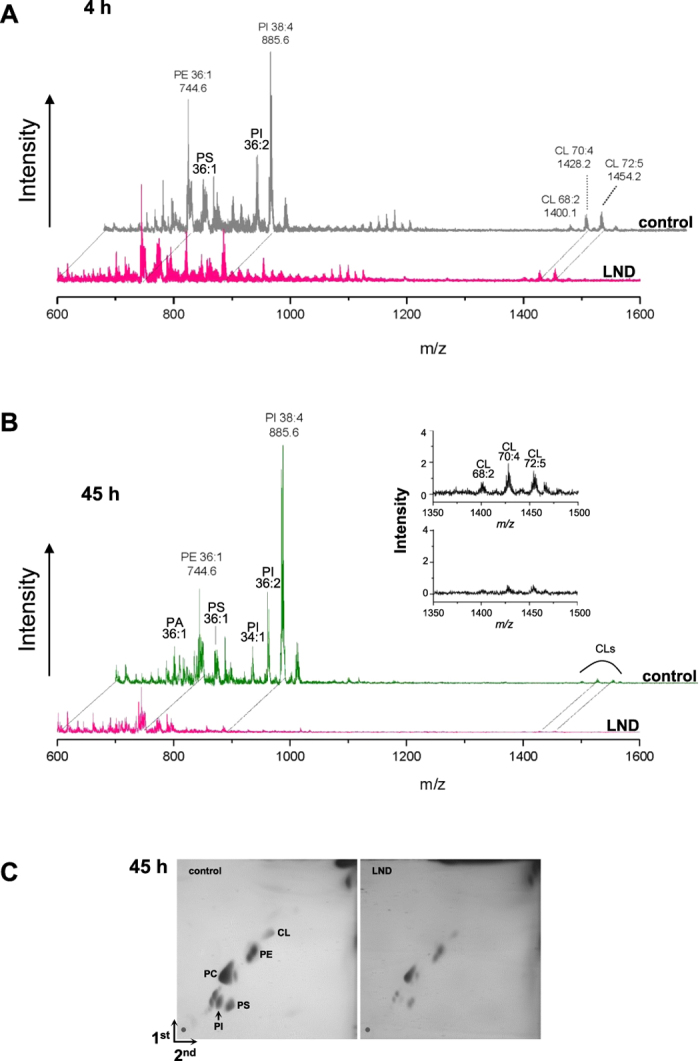
MALDI-TOF mass spectrometry lipid profiles of control and LND-treated GL-15 cells. Cells were treated with 400 μM LND for 4 hours (**A**) or 45 hours (**B**,**C**). Cells recovered from one well were subjected to lipid extraction and total lipids were analyzed by MALDI-TOF/MS (see Ref. 22) (**A,B**) or by two-dimensional TLC (**C**). In (**A,B**), the fatty acid composition of major phospholipids is indicated. In (**B**), the insert is a zoom on cardiolipins. Abbreviations are: PA, phosphatidic acid; PE, phosphatidylethanolamine; PS, phosphatidylserine; PI, phosphatidylinositol; CL, cardiolipin; PC, phosphatidylcholine.

Phospholipids constitute the primary components in membrane biogenesis during the autophagic process. Our previous work showed that 3BP treatment of GL15 cells did not affect significantly phospholipids, with the exception of a marked decrease of CL^22^. In this study the lipidomics of GL15 cells was investigated by MALDI-TOF mass spectrometry during LND-induced autophagic and post-autophagic phases (4–45 hours). The most relevant result was that phosphatidylinositol (PI) depletion occurred already at 4 hours and was complete at 45  ours of treatment. Moreover, at 45 hours the overall phospholipid content decreased but, with the exception of PI, the composition of phospholipid classes was not significantly affected (Fig. 6A,B), including phosphatidylcholine species, analyzed in ion positive mode (not shown). The phospholipid profile at 45 hours was confirmed by two-dimensional TLC analysis (Fig. 6C).

## Discussion

3BP was first introduced as a specific alkylating agent for 2-keto-3-deoxy-6-phosphogluconic aldolase and as a reactive probe in kinetic and stereochemical studies^28^. More recently 3BP has emerged as an energy blocker, thus representing a potential chemotherapeutic drug^12^. Indeed, 3BP contrasts hepatocellular carcinoma by acting preferentially against HK-II, which is highly expressed in liver carcinomas^29^. In addition, peculiar mitochondrial enzymes are also targeted by this drug^9^. New developments have extended the anticancer power of 3BP to brain tumors^22^,^30^. Shoshan recently published a state of the art review on this compound and its molecular targets^31^. Nevertheless, the biochemical mechanisms underlying the action of the drug are not yet clear. In the present study the effects produced by 3BP in GL15 glioblastoma cells were compared to the well-known antiglycolytic LND. We demonstrate that 3BP and LND direct cells to death. Both drugs induce autophagy, which constitutes the death modality in 3BP-treated cells, while in LND-treated cells autophagy eventually evolves into apoptosis, as a molecular mechanism for death.

As expected for tumor cells, the contribution of mitochondria to ATP synthesis in GL15 cells is approximately no more than 30% (Fig. 1A), the largest amount being synthesized in the cytosol. Nevertheless, the lack of its contribution channels cells to an autophagic defense (Fig. 1B). ATP levels collapsed within 1 hour of 3BP treatment, whereas sustained levels (about 60% of control) were measured for up to 40 hours of incubation with LND, the complete collapse occurring only after 65 hours (Fig. 1C,D).

The expression of autophagy-related proteins indicated that 3BP-treated GL15 cells were channeled to autophagy in 2 hours. Autophagy is a protective cellular response characterized by the conversion of LC3-I to the PE-conjugated LC3-II form that is accompanied by p62 decrease^32^, and by beclin 1 disappearance, likely by the phosphorylation and ubiquitination pathways^33^. The disappearance of the early autophagy marker beclin 1 after 2 hour incubation with 3BP suggests that the autophagic process has already evolved in the late phase, i.e. degradation^34^. At the same time, HK-II, an overexpressed protein necessary for tumor cell survival^35^, is targeted by 3BP for degradation (Fig. 2B). Previously we found that in GL15 cells 3BP activates glycogen synthase kinase-3β (GSK3β)^22^, constitutively inhibited in these cells^36^. Since the inhibition of GSK3β is involved in the association of HK-II to VDAC in mitochondria^5^,^6^,^35^,^37^, GSK3β activation could favor HK-II dissociation and proteolysis. In addition, in hepatoma cells the main blocking site of 3BP is the mitochondrial HK-II^29^, through 3BP binding to cysteins that causes HK-II dissociation from the outer mitochondrial membrane and detachment from VDAC^38^. We reported that no caspase activation, phosphatidylserine externalization, or DNA fragmentation occur after 18 hours of incubation with 3BP^22^, consistent with the persistence of an autophagic program, not overcome by apoptosis. We hypothesize that lack of the switch that overcomes autophagy could be related to cyt c and p53 disappearance. Cyt c, which is tightly bound to the inner mitochondrial membrane in GL15 cells, can be released outside the mitochondria in response to fatty acid saturation of mitochondrial CL, thus triggering caspase-3 activation and DNA fragmentation^19^. Cyt c degradation in 3BP-treated cells excludes this apoptotic cascade.

It has been demonstrated that inhibition, depletion, or deletion of the tumor suppressor p53 increases biochemical signs of autophagy^23^. Many different inducers of autophagy stimulate proteasome-mediated degradation of p53 as a mechanism enhancing an autophagic program. At the same time the inhibition of p53 degradation prevents the activation of autophagy in several cell lines in response to different stimuli^23^. Normal unstressed cells contain very low levels of p53 protein, due to continuous synthesis and degradation in a MDM2-mediated process^39^. Following stress, p53 is phosphorylated at multiple residues. In particular, the Ser-15 phospho-acceptor site is important for tumor suppression activity^40^. Once activated and phosphorylated p53 escapes from ubiquitin-dependent degradation^41^. In our study, p53 response to 3BP in GL15 cells has been compared to that in U251 and U87 glioblastoma cell lines. All glioblastoma cells express high p53 content in the phosphorylated status (Ser-15 and Ser-315), suggesting that phosphorylated p53 is preserved from degradation. In GL15, dephosphorylation of Ser-15 and Ser-315 caused by 3BP is followed by p53 degradation (Fig. 2C,D). This event does not occur in U87 and in U251, where only Ser-315 site undergoes dephosphorylation. Although the link between p53 stabilization and Ser-315 phosphorylation may not be a general rule, our finding supports the model that in GL15 cells p53 phosphorylation at Ser-15 prevents its interaction with MDM2, the protein targeting p53 for proteolysis^24^ (Fig. 2C–E).

LND did not trigger an autophagic response of GL15 cells at 2 hour treatment, but treatments as long as 48 hours resulted in concentration-dependent conversion of LC3-I to LC3-II and its disappearance. At the same time cells showed acidic vesicular organelles, LC3 in autophagosome membranes, and loss of cytoskeleton integrity (Fig. 3). Although cytoskeleton alterations could be representative of an autophagic scenario, the possibility that they could predict the induction of apoptosis should not be excluded^25^. Moreover, the increase of p62 expression (Fig. 3B) highlights the possibility of an evolution of autophagy into apoptosis^26^. Indeed, DNA fragmentation, which was not detected at 24 hours, occurred at 48 hours of LND treatment and was extensive at 72 hours (Fig. 4). Since cyt c was almost completely degraded at 48 hours (Fig. 3B) and caspase-3 was not activated at any time, cell death pathways converging on the proteolytic activation of caspase-3 can be excluded. However, p53 was not degraded but translocated from nuclei to mitochondria (Fig. 5). The p53 protein can regulate cell apoptosis by a transcription-independent pathway, involving its translocation to mitochondria, where the C-terminal region becomes tightly bound to CL^41^.

Translocation of p53 to mitochondria may be controlled by Akt, whose dephosphorylation favors translocation and apoptosis^27^,^42^. Indeed, we found that Akt phosphorylation was decreased after LND treatment of GL15 cells (Fig. 5C), which could be responsible for p53 translocation to mitochondria. When bound to mitochondria, p53 suppresses autophagy and favors the induction of mitochondrial outer membrane permeabilization to pro-apoptotic proteins^23^. Among these, AIF is a flavoprotein that translocates from mitochondria to the nucleus and causes DNA fragmentation^43^. Since AIF was not degraded (Fig. 5A), we speculate that this protein could be involved in the apoptotic event observed at 48 hours of LND treatment.

In GL15 cells, Akt is constitutively activated^20^. PIP3 is an activator of the phosphoinositide-dependent kinase (PDK1) responsible for Akt activation. At 4 hours of LND treatment PI content decreased and completely disappeared at 45 hours (Fig. 6). This could prelude to overcoming autophagy and switching to apoptosis. Indeed, the selective disappearance of PI could deplete substrate for PIP3 synthesis, thus resulting in Akt dephosphorylation and in the block of PI3K/Akt signaling. It is worth noting that in 3BP-treated GL15 cells, although no PI disappearance occurred^22^, Akt was also dephosphorylated, which could correlate with the dramatic decrease of ATP levels.

In conclusion, GL15 cells follow different death pathways, depending on the insult received. LND triggers a late autophagic response that eventually evolves into apoptotic cell death. Apoptosis is preceded by PI disappearance and pAkt decrease. Turning off the PI3K/Akt pathway could favor p53 translocation to mitochondria that triggers the p53-dependent apoptotic route. 3BP exerts very early effects leading to complete ATP depletion and aggressive autophagy involving p53 dephosphorylation and degradation that hinders the evolution towards apoptosis.

## Material and Methods

### Chemicals

Bromopyruvate (3BP), lonidamine (LND), mitochondrial inhibitors, respiratory substrates, chemicals, LC3 rabbit polyclonal antibody, α-tubulin and β-tubulin mouse monoclonal antibodies, and goat anti-mouse FITC-conjugated IgG were from Sigma (Milan, Italy). Cyt c mouse monoclonal antibody, AIF goat polyclonal antibody, β-actin goat polyclonal antibody, p53 (FL-393) rabbit polyclonal antibody, p-p53 (Ser-315) rabbit polyclonal antibody, HK-II (C-14) goat polyclonal antibody, beclin 1 (E-8) mouse monoclonal antibody, goat anti-mouse HRP-conjugated IgG, goat anti-rabbit HRP-conjugated IgG, and rabbit anti-goat HRP-conjugated IgG were from Santa Cruz Biotechnology; VDAC1 rabbit polyclonal antibody was from Calbiochem. p-p53 (Ser-15) rabbit polyclonal, SQSTM1/p62 rabbit polyclonal, Akt rabbit polyclonal, and p-Akt (Ser-473) rabbit monoclonal antibodies were from Cell Signaling Technology. LC3 mouse monoclonal antibody was from NanoTools. Mito-tracker red CM-XRos, Alexa Fluor® 488 goat anti-rabbit IgG, and Alexa Fluor® 555 goat anti-mouse IgG were Molecular Probes products.

### Cell culture and treatments

Glioblastoma cells were grown as previously described^44^. The cells were trypsinized, plated in 6-well plates (2 × 10^5^ cells per well), and incubated for three days at 37 °C in a 5% CO_2_ humidified atmosphere to obtain semi-confluent cells. Medium was changed to serum-free DMEM and cells were subjected to the following treatments. i) Incubation for 6 hours with the respiratory chain inhibitors, rotenone (10 μM), antimycin A (25 μM), valinomycin (2 μM), or CCCP (10 μM). ii) Incubation with a buffered solution of 3BP (pH 7.4) or LND for the indicated times. 3BP was used in the range of 50–80 μM^36^, whereas LND was in the range 50–400 μM^45^. The effects of 3BP and LND on relevant endpoints were analyzed by comparing 80 μM 3BP and 400 μM LND. Caspase-3 activity was determined using Ac-DEVD-AFC as substrate^19^. In selected experiments, U251 and U87 glioblastoma cell lines were used for comparison with GL15.

### ATP determination

ATP levels in cells were quantified by the ATP Bioluminescent Assay (FLAA, Sigma) by using a calibrated ATP standard curve.

### Western blot analysis

After treatments, whole cell lysates were prepared using lysis buffer (1% SDS, 1 mM Na-vanadate, 10 mM Tris-HCl pH 7.4) in the presence of 0.1 mM phenylmethylsulfonyl fluoride and protease inhibitor cocktail. Viscosity of the samples was reduced by sonication. Forty microgram protein was subjected to SDS-PAGE and electroblotting on nitrocellulose or PVDF membranes, which were probed with specific primary and HRP-conjugated secondary antibodies. Immunoblots were revealed by enhanced chemiluminescence reagent (Bio-Rad). Images were acquired using the VersaDoc 1000 imaging system and individual band densities were integrated by Quantity One software (BioRad).

### qRT-PCR

Total RNA was isolated from cells with TRIZOL Reagent (Invitrogen) according to the manufacturer’s instructions and cDNA was synthesized using cDNA synthesis kit (ImProm-II Reverse Transcription System-Promega). qRT PCR amplifications were performed using Mx3000P Real-Time PCR System with Brilliant SYBR Green QPCR Master Mix (Stratagene) and ROX as reference dye. The primers used are listed in supplementary table 1. Each sample was run in triplicate. Mean of Ct values of the samples was compared to the untreated control. HPRT was used as internal control. The n-fold differential ratio was expressed as 2^−ΔΔCt^.

### Acridine orange staining of GL15 cells

GL15 cells were grown in glass coverslips for 3 days, then serum starved for 48 hours in the presence or absence of 400 μM LND. The cells were stained with 2 μg/ml acridine orange (AO) in phosphate buffered saline for 15 min at 37 °C and immediately analyzed with a fluorescence microscope (DMRB Leika).

### Indirect immunofluorescence

Cells were grown on glass coverslips and treated as in the AO experiments. For LC3II detection, the cells were washed with PBS, fixed with 4% paraformaldehyde/PBS for 15 min, washed with PBS, and incubated in blocking solution (3% albumin/PBS + 0,2% triton X-100) for 15 min. After washing with PBS, the cells were incubated overnight at 4 °C with the anti-LC3 mouse monoclonal antibody (1:100 in blocking solution). After extensive washing with PBS, the cells were incubated for 50 min at room temperature with the secondary antibody (Alexa Fluor® 555 goat anti-mouse IgG 1:400), washed with PBS, and stained with DAPI for 5 min. For α-tubulin detection, the cells were washed with PBS, fixed with 4% paraformaldehyde/PBS for 15 min, and processed as described^44^. The dilution of the monoclonal α-tubulin antibody was 1:1000 in 0.3% albumin/PBS. The procedure for p53 localization was performed after loading the cells with 500 nM Mito-tracker red at 37 °C for 15 min. After washing with PBS, the cells were fixed with 4% paraformaldeide/PBS for 15 min, rinsed in PBS, permeabilized with 0.1% triton X-100/PBS for 10 min, and washed in PBS. After 30 min of incubation in 1% albumin in PBS, the cells were incubated overnight at 4 °C with the anti-p53 polyclonal antibody (1:150 in 1% albumin/PBS). The subsequent steps were performed as described^44^, with the exception that the secondary antibody was Alexa Fluor® 488 goat anti-rabbit IgG (H + L) (1:1000 in 1% albumin/PBS).

### Flow cytometry analyses

To evaluate plasma membrane integrity after 3BP treatment, U87 and U251 cells were resuspended in isotonic buffer and stained with Annexin V-FITC (1 μg/ml) plus propidium iodide (1 μg/ml) using the Annexin V Apoptosis Detection Kit FITC (Affymetrix), and analyzed by flow cytometry, using a FACScan flow cytometer (Beckman Coulter Epics XLMCL) equipped with a focused argon laser. Apoptotic index of LND-treated GL15 and U251 cells was determined by flow cytometry analysis of fragmented DNA after propidium iodide staining (1 μg/ml) in hypotonic solution. For each sample, 10,000 events were recorded and cells with a hypoploid DNA content were quantified as apoptotic cells.

### MALDI-TOF mass spectrometry

Total lipids of cells derived from one well were extracted and analyzed both in ion negative and ion positive mode as previously described^22^,^46^. Alternatively, phospholipid classes were separated by two-dimensional TLC and lipids were visualized with Cu-acetate reagent^22^.

### Statistical analyses

The results, expressed as means ± SD of at least three independent experiments, were analyzed for statistical significance by Student’s *t*-test. p-values < 0.05 were considered significant.

## Additional Information

**How to cite this article**: Davidescu, M. *et al*. The energy blockers bromopyruvate and lonidamine lead GL15 glioblastoma cells to death by different p53-dependent routes. *Sci. Rep*. **5**, 14343; doi: 10.1038/srep14343 (2015).

## Supplementary Material

Supplementary Information
