# Supplementary material for: The energy blockers bromopyruvate and lonidamine lead GL15 glioblastoma cells to death by different p53-dependent routes
Source: Sci Rep. 2015 Sep 21;5:14343. doi: 10.1038/srep14343 (PMC4585687; doi:10.1038/srep14343)
Supplement: Supplementary Information [file srep14343-s1.doc]

**The energy blockers bromopyruvate and lonidamine lead GL15 glioblastoma cells to death by different p53-dependent routes**

Magdalena Davidescu^a#^, Lara Macchioni^a#^, Gaetano Scaramozzino^b^, Maria Cristina Marchetti^c^, Graziella Migliorati^c^, Rita Vitale^d^, Angela Corcelli^e^, Rita Roberti^a^, Emilia Castigli^b§^, Lanfranco Corazzi^a§*^

**^a^**Department of Experimental Medicine, University of Perugia, Perugia, Italy. **^b^**Department of Chemistry, Biology and Biotechnology, University of Perugia, Perugia, Italy. **^c^**Department of Medicine, University of Perugia, Perugia, Italy. **^d^**IMM-CNR, Institute for Microelectronics and Microsystems, National Research Council, Lecce, Italy. **^e^**Department of Basic Medical Sciences, Neuroscience and Sensory Organs, University of Bari “A. Moro”, Bari, Italy.

*Corresponding author: Lanfranco Corazzi, Department of Experimental Medicine, Section of Physiology and Biochemistry, University of Perugia, 06132 Perugia, Italy. Tel.: 39 075 5858196; e-mail: lanfranco.[corazzi@unipg.it](mailto:corazzi@unipg.it)

**Supplementary t**able 1. List of primers

| **Gene name** | **Gene symbol** | **Primer sequence** | |
| --- | --- | --- | --- |
| Cytochrome c | CYCS | | *For*:TGAAAAGGGAGGCAAGCACA  *Rev*:TCCTCTCCCCAGATGATGCC |
| Beclin-1 | BECN1 | | *For*:CCGAGGGATGGAAGGGTCTA  *Rev*:CTCGTGTCCAGTTTCAGGGG |
| Hexokinase II | HK2 | | *For*:GCCTAGATGACTTCCGCACA  *Rev*:AGATGCCCCTTGTCTTGAGC |
| p53 | p53 | | *For*:TGACACGCTTCCCTGGATTG  *Rev*:GCTCGACGCTAGGATCTGAC |
